# The Incremental Role of Coronary Computed Tomography in Chronic Coronary Syndromes

**DOI:** 10.3390/jcm9123925

**Published:** 2020-12-03

**Authors:** Francesca Baessato, Marco Guglielmo, Giuseppe Muscogiuri, Andrea Baggiano, Laura Fusini, Stefano Scafuri, Mario Babbaro, Rocco Mollace, Ada Collevecchio, Andrea I. Guaricci, Mark Rabbat, Antonello D’Andrea, Gianluca Pontone

**Affiliations:** 1 Department of Cardiology, San Maurizio Regional Hospital, 39100 Bolzano, Italy; francesca.baessato@sabes.it; 2 Centro Cardiologico Monzino IRCCS, 20138 Milan, Italy; marco.guglielmo@ccfm.it (M.G.); giuseppe.muscogiuri@ccfm.it (G.M.); andrea.baggiano@cardiologicomonzino.it (A.B.); laura.fusini@ccfm.it (L.F.); stefano.scafuri@ccfm.it (S.S.); mario.babbaro@ccfm.it (M.B.); rocco.mollace@ccfm.it (R.M.); 3 Department of Cardiac, Thoracic, Vascular Sciences and Public Health, University of Padua, 35128 Padua, Italy; ada.collevecchio@gmail.com; 4 Department of Emergency and Organ Transplantation, Institute of Cardiovascular Disease, University Hospital Policlinico of Bari, 70124 Bari, Italy; andrea.guaricci@gmail.com; 5 Center for Heart & Vascular Medicine, Loyola University Medical Center, Maywood, IL 60153, USA; mrabbat@lumc.edu; 6 Edward Hines Jr. VA Hospital, Hines, IL 60141, USA; 7 Department of Cardiology, Umberto I Hospital, 84014 Nocera Inferiore, Italy; antonellodandrea@libero.it

**Keywords:** coronary computed tomography, coronary calcium score, fractional flow reserve, perfusion imaging

## Abstract

In the context of chronic coronary syndromes (CCS), coronary computed tomography angiography (CCTA) has gained broad acceptance as a noninvasive anatomical imaging tool with ability of excluding coronary stenosis with strong negative predictive value. Atherosclerotic plaque lesions are independent predictors of cardiovascular outcomes in high risk patients with known coronary artery disease (CAD). Calcium detection is commonly expressed through the coronary artery calcium score (CACS), but further research is warranted to confirm the powerness of a CACS-only strategy in both diagnosis and prognosis assessment. Recent studies evidence how defined plaque composition characteristics effectively relate to the risk of plaque instabilization and the overall ischemic burden. Fractional flow reserve from CCTA (FFR-CT) has been demonstrated as a reliable method for noninvasive functional evaluation of coronary lesions severity, while the assessment of perfusion imaging under stress conditions is growing as a useful tool for assessment of myocardial ischemia. Moreover, specific applications in procedural planning of transcatheter valve substitution and follow-up of heart transplantation have gained recent importance. This review illustrates the incremental role of CCTA, which can potentially revolutionize the diagnosis and management pathway within the wide clinical spectrum of CCS.

## 1. Introduction

Chronic coronary syndromes (CCS) are a widespread phenomenon associated with different clinical entities, mostly characterized by a stable and progressive process of atherosclerotic plaque formation. As symptoms among patients with CCS are often not uniform and atypical, diagnostic tests are needed to confirm the diagnosis and evaluate the risk of events. Over the past years, several noninvasive imaging methods have evolved as the “gatekeeper” for invasive coronary angiography (ICA), like positron emission tomography (PET), stress-echocardiography, single-photon emission CT (SPECT), and stress cardiac magnetic resonance (CMR).

Coronary CT angiography (CCTA) has been validated as a noninvasive anatomical imaging test with an affordable safety profile and diagnostic accuracy in excluding the presence of coronary stenosis [1,2,3]. Rapid developments in scanner technology and recent scientific evidence have raised a great potential for CCTA beyond coronary imaging, as a promising risk stratifier in patients with coronary artery disease (CAD) [4,5]. The possibility of providing detail about coronary anatomy, plaque morphology, disease activity, and hemodynamic effects of coronary lesions in a single examination has made CCTA an appealing noninvasive diagnostic modality for the assessment of CCS.

## 2. State of the Art: Technology

### 2.1. Evolution of Cardiac CT Scanners

The first developed electron beam CT scanners, useful for coronary calcification imaging, have been replaced by multidetector CT scanners, which have rapidly developed since the 1980s, for adequate performance of contrast-based cardiovascular imaging such as CCTA. Today, a 64-detector rows technology is required for CCTA studies [6], although calcium scoring systems with 16 detector rows may be sufficient.

In 1998, the “4-detector-row” scanners were introduced in clinical practice for imaging the coronary arteries, although these early models had limited applicability and mainly confined to coronary calcium scoring [7]. Coronary angiography became then feasible with the use of 16-detector-row CT scanners and with retrospective cardiac gated acquisitions [8]. A greater improvement in the diagnostic accuracy was later reached with the introduction of 64 detector rows, with faster gantry rotation times (350–420 ms) and reduced detector size (0.5–0.65 mm). However, the early 64-slice multi-detector CT scanners (MDCT) showed an inherent limited temporal and spatial resolution, with a restricted *z*-axis coverage. These were rapidly overcome by newer generation MDCTs, which presented improved spatial and temporal resolution, faster scan mode, and whole heart coverage with either wide-detector or dual-source CT. Wide-detector CT scanners with a wide area-coverage (16 cm) can acquire images of the whole heart within one heartbeat, without stair-step artifacts [9]. Table 1 provides an overview on multi-slice CT technology with details on progressive improvement on both spatial and temporal resolution.

Dual-source CT has high-pitch acquisition platforms that can image the heart in less than 300 ms. Furthermore, the use of thinner detectors with a spatial resolution of 250 microns along the XY planes, faster gantry rotation times (220 ms), noise reduction stretegies with improved detector efficiency, and new electronic circuitry have allowed diagnostic image quality in patients who were considered to be challenging with prior-generation CT. For example, patients with calcium scores >400 HU, coronary artery stent <3 mm, coronary artery bypass grafts, significant heart rate variability and heart rates >65 bpm, and body mass index >30 kg m^2^ could now be adequately scanned [10,11,12].

### 2.2. Radiation Exposure and Principle Cardiac CT-Related Risks

Main risks related to CCTA include radiation exposure and contrast agent nephropathy. A significant reduction in total radiation dose as well as contrast material volume has been recently achieved thanks to technological advances.

Patients at higher risk for contrast-induced nephropathy are those with reduced renal function (eGFR <45 mL/min/1.73 m^2^) and with diabetes, especially when both present [13]. Therefore, since a significant proportion of patients with CCS are at risk for contrast-induced nephropathy, optimization of the contrast material dose is essential, though allowing a proper magnification of the vessel lumen [14]. The latest CT scanners can allow a very short acquisition time, therefore requiring a markedly reduced contrast material volume for coronary opacification. For example, 75–100 mL are now sufficient for 64-detector-row CT systems, while 100–140 mL were originally needed with 16-detector rows. [15]. Even less than 60 mL may be appropriate for newer wide-detector CT scanners [16,17]. Iterative reconstruction techniques and low tube potential (70–100 kV) have also favored a reduction in contrast dose. However, in order to allow an accurate delineation of coronary disease, attenuation values between 250 and 350 HU should be guaranteed [18,19].

Dose reduction strategies are currently needed to limit patient exposure, especially with the introduction of multi-detector CT [20]. In regard to dose reduction strategies, axial volumetric acquisition of CCTA may reduce the overall radiation dose without additional radiation exposure derived from helical oversampling or sequential axial scanning [21]. For example, an effective dose of 8.3 ± 3.4 mSv was reported for 320-detector-row CT scanners, due to tube current and X-ray emission modification based on the patient’s body size [22]. Several years later, an even lower radiation dose, till less than 1 mSV, was achieved with faster gantry rotation times and other technical improvements, though providing an excellent image quality over a wide range of body sizes and heart rates at lower radiation dose [23,24]. Table 2 underlines the different radiation exposure ranges for different CT modalities. Of note, FFR-CT does not add further risk to standard CCTA.

Other risks in CT examinations may concern additional contrast agent-related effects, allergic reactions, and medications side effects. Severe allergic reactions are rare (0.04%) [25]. Contrast medium extravasation is relatively rare (frequency <1%) and generally results in minimal damage at superficial skin level, although it may eventually lead to severe stages of necrosis and ulceration which may also favor topic infection [26]. Beta-blockers should be used with caution in patients with asthma or bronchospastic disease, atrio-ventricular conduction defects, and should not be administered if systolic blood pressure <90 mmHg. Care should regard administration of nitroglycerine in aortic stenosis patients, and it should be avoided with phosphodiesterase type 5 inhibitors and hypertrophic cardiomyopathy [27].

### 2.3. Future Technical Perspectives

Some future perspectives in the field of cardiac CT hardware technology include the use of true cardiac-capable photon counting detectors, which enable nearly 100% geometric dose efficiency, permitting relevant radiation dose reduction [28,29] with far superior spatial resolution, giving way to improved coronary lumen visualization through better edge delineation [30]. These advances may also offer the ability to perform advanced tissue analysis with distinction of lipid, fibrous, and calcified elements [31].

Apart from advancements in hardware technologies, the integration of validated advanced analytic tools and engineering solutions, such as machine learning and deep learning, is promisingly leading to an innovative and impressive pathway in study analysis and reporting. Early experience has outlined the emerging role of these analytical tools in CT datasets to unmask their new potential compared with standard visual evaluation [32]. These techniques could significantly overcome limitations related to human interpretation, with its inherent limitations, with a rapid and objective image dataset evaluation. Development of these complex algorithms requires highly advanced mathematics, engineering, and computer programming levels, which are still under construction and evolving. These tools may represent an increasing part of routine clinical practice in the future, but further large randomized trials are necessary to validate these innovative approaches.

## 3. Screening of Patients: The Calcium Score

Coronary artery calcification shows a significant association with advanced coronary disease burden [33], and represents a final step of progression for coronary plaque [34]. Various techniques are used to detect the amount of calcium deposits, including the Agatston calcium score. This score was first developed in the 1990s, being the most broadly used method to detect coronar artery calcification, as it shows a discrete reproducibility and accuracy.

Coronary artery calcium score (CACS) is commonly achieved in the initial noncontrast low-radiation phase of CCTA, by assigning a weighted density score to the location of calcium with the highest attenuation (measured in Hounsfield units) and then multiplying by the area of calcification. It is defined as >130 HU and >1 mm^2^ in size. In detail, grading of coronary calcium burden is defined as a 0, 1 to 10, 11 to 100, 101 to 400, and greater than 400 CACS, which correspond to no, minimal, mild, moderate, and severe CAD [35].

Numerous studies have outlined the prognostic value of CACS over conventional risk factors in asymptomatic patients with a pre-test intermediate risk of cardiovascular disease (CVD). Approximately 20% of patients in the intermediate risk group will have an improvement in risk prediction when CACS is considered (6–20% risk for events in the next 10 years according to the Framingham risk score). The lowest risk of adverse events is in the 1–100 group (HR 3.61), with the highest in the >400 group (HR 9.67) [36]. A low prevalence of obstructive disease is related to an Agatston = 0, with a <1% annual risk for nonfatal myocardial infarction, providing a “warranty” against cardiovascular events for 10–15 years [37,38].

The recent 2019 European Society of Cardiology (ESC) guidelines for CCS give CACS a Class IIb recommendation as a screening tool for CAD in asymptomatic patients, while the current 2016 European guidelines on preventive strategies give CACS a Class IIa recommendation for intermediate risk patients [39]. CACS, along with several CVD risk factors, namely age, sex, ethnicity, diabetes, tobacco use, cholesterol level, blood pressure, and use of cholesterol or hypertensive medications can provide an adequate predictive model of 10-year-risk for CVD events [40,41]. Recent studies have therefore supported the integration of CACS into CVD risk prediction models above traditional risk factors. For example, the Astronaut Cardiovascular and Health Modification (Astro-CHARM) calculator demonstrated the improvement of adding CACS to the Framingham Risk Score [42]. The Multi-Ethnic Study of Atherosclerosis demonstrated that CACS predicted CVD events beyond traditional risk factors with adequate strength in all ethnic groups represented in the study [43]. Calcium scoring is also considered a decisive factor in the decision to begin statin therapy as recently incorporated into the 2018 US guidelines for the management of blood cholesterol [44]. For example, a CACS of 0 generally supports deferral of statin therapy unless a patient has diabetes, is a cigarette smoker, or a family history of premature coronary disease. Otherwise, a CACS of 1–99 favors statin therapy [45].

On the other hand, CACS seems to be less useful in low-risk patients. In addition, high-risk or symptomatic patients do not benefit from this examination [46]. One of the major benefits of CACS is in the reclassification of intermediate risk patients, who are not symptomatic, into a higher or lower risk group, thus identifying those who may benefit more from an aggressive primary preventive treatment, based on the very favorable prognosis of a CACS of zero [45,46,47]. CACS may therefore be useful to adjust the pharmacologic therapy and adapt lifestyle modifications in order to provide targeted risk factors modifications, in particular treatment of hypertension, dyslipidemia, and diabetes. CACS can be considered for CAD population screening due to its little radiation exposure and need for patient preparation. Of note, seriated CACS performance would easily help to monitor vessel disease progression and/or regression, though with limited information than CCTA.

However, certain aspects should be taken in consideration. For instance, traditional CACS cannot provide the number and size of calcifications, thus limiting comprehensive assessment of total plaque burden, which is considered an important feature of adverse event risk. In addition, CACS alone is not able to identify noncalcified coronary artery plaques, which represents a large portion of total plaque content, thus significantly underestimating and missing a part of atherosclerotic pathology [48,49]. If histological data suggest that plaques with high calcium amount have smaller lipid cores and less positive remodeling, which are defined features of vulnerable plaques, CACS actually targets a stable type of plaque, which is less susceptible to rupture or cause adverse events.

Although current recommendations for CACS performance in routine clinical practice remains, future and rapidly evolving advances in plaque imaging as well as newer improvements in CT technology will likely overcome the role of CACS as a screening tool for CAD in the future.

## 4. Clinical Indications of CCTA in the Context of Chronic Coronary Syndromes

The 2019 European Society of Cardiology (ESC) guidelines on the diagnosis and management of CCS [50] introduced the term CCS, which refers to a defined spectrum of clinical scenarios of CAD, without including acute coronary events.

Patients suspected of CCS should be managed through a stepwise approach, to select the most appropriate noninvasive functional or anatomical modality from clinical patient’s characteristics (gender, age, and symptoms) so as to assess the risk of obstructive CAD and possibly intervene with revascularization. A pre-test probability (PTP) <5% does not suggest further testing, while in patients with PTP >15% or PTP between 5–15% and a strong clinical suspect of obstructive CAD, a subsequent noninvasive diagnostic test should be performed [51]. Only if the assessed risk of obstructive CAD results is very high, ICA should be recommended.

The choice for noninvasive diagnostic testing in patients with intermediate PTP of CAD may depend on local availability, expertise, and patient’s characteristics. However, current 2019 ESC guidelines on CCS recommend CCTA as the first-line test (class IB) in suitable patients with low to intermediate clinical likelihood of CCS, due to its highest rule-out capability compared to other noninvasive tests. This gives a growing role to CCTA, representing a dramatic change in respect to the previous version of the guideline. Otherwise, functional imaging tests such as stress-echocardiography, PET, SPECT, or CMR perfusion imaging may be preferred in certain healthcare settings mainly due to local availability or as an alternative of CCTA when doubtful or not diagnostic, as well as in patients with known CAD or who have undergone previous percutaneous coronary intervention (PCI) [50].

The SCOT-HEART (Scottish Computed Tomography of the Heart) trial randomized 8000 patients with suspected obstructive CAD to either CCTA or standard care (predominantly stress ECG), thus revealing a significantly lower rate of the primary endpoint of cardiovascular death or nonfatal MI (2.3% vs. 3.9% at 5-year follow-up) in patients who underwent CCTA [52,53]. Rates of ICA and revascularization did not differ significantly between the two strategies, CCTA led to the beginning of more preventive therapies [53].

The PROMISE (Prospective Multicenter Imaging Study for Evaluation of Chest Pain) trial randomized 10,003 patients with symptoms suggestive for CAD randomized to either CCTA or functional tests, without showing any relevant difference in the primary outcome (3.3% vs. 3.0%, at 25-month follow-up) [54].

In the EVINCI (Evaluation of Integrated Cardiac Imaging in Ischemic Heart Disease) study, CCTA was compared with several diagnostic modalities (stress CMR, PET, SPECT, stress echocardiography) in patients with suspected CAD and likelihood of intermediate disease. In this study, the CT scan was found to be the method with the best diagnostic performance (sensitivity, specificity, and diagnostic accuracy of 91%, 92%, 91%, respectively) [55]. Moreover, other randomized trials documented that CCTA performs exclusion of CAD in a rapid, safe method, with a very low rate of complications related to contrast medium (<1/1000 patients), and with similar or superior cardiac results compared to noninvasive functional testing [52,54,55,56].

**Figure 1:**
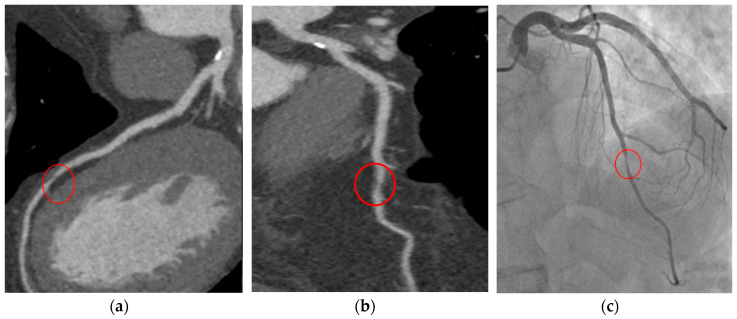
T.F. 67 years-old, male; dyslipidaemia, smoker; typical exertional chest pain; exercise test negative; CCTA (**a**,**b**): subocclusive stenosis of mid-LAD; ICA (**c**): significant stenosis was confirmed and elective PCI was performed. CCTA: coronary computed tomography angiography; LAD: left anterior descending artery; ICA: invasive coronary angiography; PCI: percutaneous coronary intervention.

CCTA shows very high sensitivity in detecting both coronary artery stenosis defined as obstructive by ICA and non-obstructive calcified or non-calcified lesions [56]. Meta-analysis assessing the diagnostic performance of CCTA in respect to ICA (for >50% coronary stenosis) demonstrated an overall sensitivity and specificity of 96.6% and 81.5%, respectively [57]. Although CCTA may be associated with an increasing number of total ICAs, it allows a reduced percentage of negative ICA studies performed, as well as myocardial infarctions rate, with more appropriate revascularizations in respect to functional tests [58]. The favorable long-term outcome may be favored by the reliable ability in the identification of CAD and the subsequent begin of preventive therapies [59]. Figure 1 is an example of CCTA acquisition and subsequent ICA in the same patient.

CCTA can also provide important prognostic information [60] and may allow risk stratification and guide future therapy decisions in CAD patients [37,59]. The culmination of recent advances in the field of CCTA has favored a change in the latest National Institute for Health and Care Excellence (NICE) guidelines for recent onset chest pain, which highly recommend CCTA for patients with typical or even atypical nonanginal chest pain with ECG changes [61]. This gives CCTA a more relevant role in the diagnosis of CAD in respect to calculations of pre-test probability. An updated version of the American guidelines in the context of stable CAD is expected in the near future, which will probably further define the cardiac CT role among CCS patients. According to existing guidelines, CCTA is not recommended in the presence of adverse features that could compromise good image quality, such as diffuse coronary calcifications, irregular heart rates, obesity, and difficulty in breath-holding [50]. However, growing technologies already permit acquisition of good image quality in patients historically considered not suitable for this examination. Thus, this recommendation could potentially be overcome in the next few years, further amplifying the already wide field of application for CCTA.

## 5. Prognosis and Risk Stratification: Plaque Imaging

**Figure 2:**
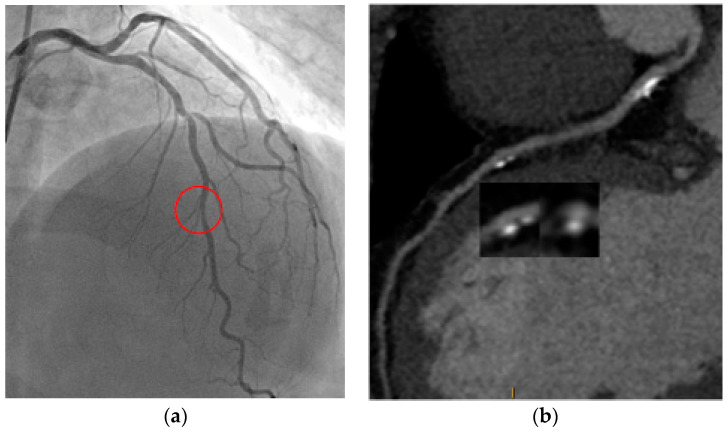
S.M. 54 years-old, male; dyslipidaemia, smoker, PAD; shortness of breath; CCTA (**a**): high risk plaque feature in left descending artery with spotty calcifications, positive remodeling; ICA (**b**): significant tandem-stenosis of LAD. PAD: peripheral artery disease; CCTA: coronary computed tomography angiography; ICA: invasive coronary angiography; LAD: left anterior descending artery.

Thanks to recent technological developments, CCTA has become a reliable tool not only in delineating anatomically the coronary arteries, but also in allowing a comprehensive clinical evidence of coronary plaques (Figure 2).

In respect to ICA, CCTA allows direct atherosclerotic plaque visualization in a rapid, noninvasive way, overcoming the need for invasive catheter tools. Prompt referral for ICA in CAD patients is mainly guided by diameter stenosis, which is the most clinically validated element to guide revascularization [62]. However, although the risk of plaque rupture is proportional to the degree of stenosis, it was documented how the majority of culprit lesions found in acute coronary syndromes (ACS) are caused by nonobstructive lesions but associated with typical features of plaque composition [63]. Therefore, integration of plaque characteristics may lead to improved prediction of adverse events.

Lesions at higher risk for acute instability are known as “vulnerable” plaques [64]. The first insight came from histopathologic studies, which defined typical features of vulnerable plaques, such as large necrotic core, thin fibrous cap, plaque and perivascular inflammation, positive remodeling, and spotty calcification [65,66]. Narula et al. defined the fibrous cap thickness as the best predictor of vulnerable plaques [67]. On CCTA, thin-cap fibroatheromas are noninvasively identified with the “napkin ring” sign. This sign corresponds to a low-attenuation core, with a thin hyperattenuated ring around it [68], and has been shown as independent predictor of future cardiac events (HR 5.55, *p* < 0.001) [69].

Apart from fibrous cap thickness, a sub-analysis of Narula et al. on histopathologic studies, identified macrophage cells presence and necrosis extension in the plaque core as other important features that favor plaque rupture [67]. Novel tracers can be used in hybrid imaging technology (PET/CT) to target specific plaque molecular entities, such 18 F-sodium fluoride. This method unifies the high spatial resolution capacity and the anatomical detail of CCTA with the molecular evidence of lesion activity provided by PET. For example, 18 F-sodium fluoride uptake corresponded to different adverse elements on standard CT [70].

Perivascular adipose tissue may additionally represent an emerging technique for identifying areas of plaque instability. Since coronary artery disease seems to alter the composition of the adjacent adipose tissue, this effect can result in subtle changes in CT attenuation [71].

The necrotic core can be adequately visualized with CCTA, and low-density attenuation values (<30 HU as cut-off) inherent to plaque core seem to distinguish plaques with a predominant lipid-rich core [72].

Positive remodeling (remodeling index > 1.1) and low-attenuation plaques have also been identified as high-risk features on CCTA, due to inflammation grade and a thin fibrous cap [73]. Positive remodeling is related to compensatory mechanisms of coronary autoregulation, which maintain a stable vessel area when even atherosclerotic plaques extend beyond 40% of the total lumen [74]. Hoffman et al. reported a higher prevalence of remodeling in unstable plaques in respect to stable plaques among CAD (*p* = 0.04) [75].

Similarly, low-attenuation plaques identified on CCTA were most frequently related to the presence of ischemia, regardless of severity of stenosis in the NXT (Analysis of Coronary Blood Flow Using CT Angiography: Next Steps) study [76]. This association between anatomy and the pathophysiological process leading to ischemia may be related to the fact that low-density plaques more easily cause oxidative stress and inflammation locally, altering the balance between vasoconstrictor and vasodilator factors [77]. Moreover, both positive remodeling and low-attenuation plaques were strong predictors of future events (*p* < 0.001) as outlined by Motoyama et al. in a large longitudinal study [78].

Plaque consistency can be subclassified on the presence and size of calcifications (<3 mm in size defined as “spotty” vs. “large”). In large studies, spotty calcifications (87% prevalence), positive remodeling (79% prevalence) and low-attenuation core (63% prevalence) have been documented to be significantly more present in acute than stable CAD (*p* < 0.001), while, large calcifications were less frequent (*p* = 0.004) [79].

Recent large trials have confirmed the importance of high-risk plaque features in prognosis and risk assessment. A sub-analysis of the PROMISE trial in 4415 patients undergoing CCTA, high-risk plaque characteristics detected on CCTA could predict accurately future adverse events (HR 2.73) [37], and a sub-analysis of the SCOT-HEART trial revealed a higher primary composite endpoint when vulnerable plaque features were present (HR 3.01) [80].

Given this robust evidence, the Coronary Artery Disease Reporting and Data System (CAD-RADS™) guidelines recommend to report the presence of vulnerability if at least 2 of high-risk features are present in the CCTA study [81].

More recently, interest has grown on the development of quantitative, thus reproducible, imaging biomarkers, evolved from digital approaches [82]. Quantitative biomarkers have been investigated in different clinical scenarios, such as in the prediction of aemodynamically significant stenosis and plaque instabilization, therapy efficacy. Sophisticated semi-automated systems are available to quantify plaque characteristics. These are still limited to research tools, but may become routine clinical practice in the future, potentially promoting the diffusion of these innovative molecular elements in coronary imaging [83]. A post hoc analysis of the NXT trial extracted different quantitative parameters from CCTA plaques [84]. The amount of low-attenuation noncalcified plaque was a predictor of myocardial ischemia detected by invasive FFR, independently from the degree of coronary stenosis. More recently, the investigators of the CAPIRE (Coronary Plaque Features on CTA Can Identify Patients at Increased Risk of Cardiovascular Events) study supported the role of advanced atherosclerosis quantification and evaluation by CCTA in the context of extensive CAD, independently from the presence of significant coronary stenosis and high clinical risk [85,86].

In regard to disease progression, many longitudinal trials have assessed the role of quantitative coronary computed tomography (QCCTA) in the natural history of CAD through seriated CCTA studies. In the PARADIGM (Progression of AtheRosclerotic PLAque DetermIned by Computed TomoGraphic Angiography Imaging) registry, variations in QCCTA biomarkers were surrogate indicators for disease evolution [87]. The effects of intensive therapeutic regimens were also used to assess changes in coronary plaque burden in serial CCTAs in a 24-month follow-up period in 147 patients [88].

In conclusion, assessment of plaque morphology with identification and evaluation of high-risk plaque features by CCTA can help in predicting future adverse outcomes, the progression of CAD, and/or the response to management.

## 6. Advanced Techniques for Evaluation of Myocardial Ischemia: FFR-CT and Stress-CTP

Some factors limit the specificity and positive predictive value of CCTA. Accuracy is reduced in the quantification of coronary stenosis, in particular, in discriminating the potential of intermediate stenosis (coronary lumen reduction of 50–70%) to induce myocardial ischemia, a very important prerequisite in planning the therapeutic strategy (medical therapy and/or revascularization) [89]. FFR-CT analyses can clarify the hemodynamic relevance of specific lesions identified at the anatomical scan, while stress computed tomography perfusion (CTP) can unmask the presence of myocardial ischemia, with a reasonable increase in radiation exposure [90,91]. The updated NICE guidelines have endorsed the CT/FFR-CT pathway, specifying that this technique, in light of the available scientific evidence, is safe, characterized by high diagnostic accuracy, and cost-efficient [61]. Currently, no specific recommendations on the use of these emerging modalities were given in the 2019 ESC guidelines, but they both have the potential to revolutionize the diagnostic pathway of CCS.

### 6.1. FFR-CT

The FFR, first introduced in the late 1990s, is the current reference for hemodynamic assessment of coronary stenosis, and is defined by the ratio of flow with and without epicardial coronary stenosis under vasodilatory stress conditions (mostly adenosine). This corresponds in clinical practice to the invasive calculation of pressure distal and proximal to the coronary lesion [92]. If FFR <0.80 revascularization is appropriate, since it improves the prognosis of patients compared to optimal medical therapy alone, leading to a significant value in the planning of revascularization for stable coronary disease [93,94].

**Figure 3:**
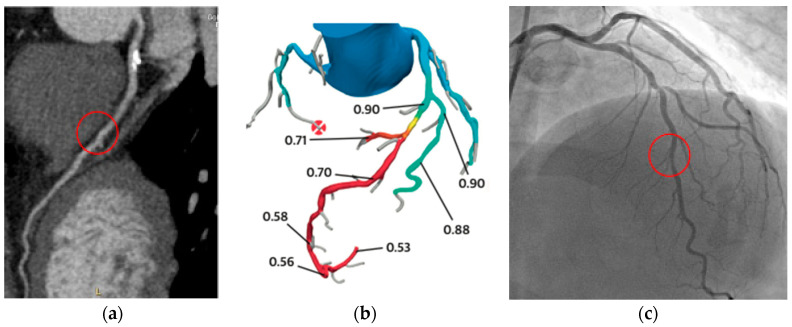
S.M. 54 years-old, male; dyslipidaemia, smoker, PAD; breath shortness; CCTA (**a**): moderate mid-LAD stenosis (see also Figure 2); FFR-CT was performed (**b**): pressure drop after diagonal branch; ICA (**c**): significant tandem-stenosis of LAD (left anterior descending artery). CCTA: coronary computed tomography angiography; LAD: left anterior descending artery; FFR-CT: fractional flow reserve derived from coronary computed tomography; ICA: invasive coronary angiography.

Recently, the FFR-CT technique has been developed to allow acquisition of functional data similar to those obtained through ICA after administration of adenosine. The FFR-CT technique permits the noninvasive calculation of FFR values for all major epicardial coronary arteries derived from the anatomical datasets of CCTA images and applying computational flow dynamics algorithms, with FFR-CT computed calculation under simulated hyperemic flow [94,95]. An example of FFR-CT is shown in Figure 3.

This analysis constructs a three-dimensional representation of the coronary tree from the acquired image dataset, and through complex fluid dynamic algorithms, the behavior of the coronary circulation under conditions of maximal hyperemia [96]. To solve these complex algorithms, it is necessary to use dedicated computers, generally off-site. One commercial software is currently approved for clinical use (HeartFlow Inc., Redwood, CA, USA). “Onsite” FFR-CT calculation systems have been recently proposed but are not commercially available for clinical practice [97]. Importantly, FFR-CT does not require additional scan data, is associated with fast processing times, without the need for a pharmacological stressor, and with a complete “offline” analysis, so that patients are not required to undergo any additional tests [98].

FFR-CT was validated against invasive FFR in three different multicenter studies, which applied similar cut-off for lesion significance (<0.80 for both modalities). Sensitivity and specificity of FFR-CCT were 86% and 79%, respectively, with significant increase of diagnostic accuracy provided by the combination of both anatomical and functional imaging. Moreover, in the subgroup of highly calcified coronary lesions, which generally overestimate stenosis by cardiac CT, FFR-CT significantly reduced the number of false positives [99,100].

The PLATFORM (Prospective LongitudinAl Trial of FFRct) study was a multicenter prospective study involving 584 patients suspected for stable CAD, who were randomized to either a CCTA + FFR-CT-driven strategy or to the conventional strategy. When FFR-CT was added to CCTA, a significant reduction of approximately 61% of ICA was gained, with equal effect on 1-year prognosis [101]. In addition, the CCTA + FFR-CT approach was cost-effective, thus significantly reducing overall costs for the healthcare system [102].In the RIPCORD (Does Routine Pressure Wire Assessment Influence Management Strategy at Coronary Angiography for Diagnosis of Chest Pain?) study, in about 36% of patients, the choice of the treatment strategy (optimal medical therapy, coronary angioplasty, aortocoronary bypass, need for additional tests) was changed, showing the relevance of a combined approach, incorporating both anatomical and functional data. In detail, 29.5% of stenoses judged severe from an anatomical point of view had a normal FFR-CT, and 4.6% of cases judged anatomically non-significant showed an abnormal FFR-CT value [103]. In the case of FFR-CT values between 0.70 and 0.80, the diagnostic accuracy was shown to be less robust than with FFR-CT values >0.80 or <0.70; this is mostly due to the lower prevalence of subjects with FFR-CT values between 0.70 and 0.80 in studies directly comparing the invasive and noninvasive FFR, and many studies incorporated older generation software algorithms [104].

The ADVANCE study is a multicenter, large prospective registry that enrolled 5083 patients referred for a CT scan investigation for suspected stable CAD in a real-world setting. In this registry, all patients also received an FFR-CT analysis, and in two out of three patients, the use of the FFRCT led to a change in the clinical management adopted. In the presence of a normal FFR-CT value, there was an important reduction in ICAs number, revascularizations, and adverse clinical events (heart attacks and deaths) at 90 days [105]. Rabbat et al. studied 431 patients who underwent a CCTA alone vs. CCTA + FFR-CT diagnostic pathway and demonstrated the safe deferral of ICA in patient with stable CAD who underwent the CCTA + FFR-CT strategy. In >90% of the cases, CCTA results were well interpretable. The majority of patients referred for ICA received a coronary stent, showing a great optimization of all invasive procedures, without increasing adverse events [106].

Recently, the SYNTAX III (Synergy Between Percutaneous Coronary Intervention with Taxus and Cardiac Surgery) Revolution study demonstrated that a “heart team” consisting of a cardiologist expert in CCTA imaging, an interventional cardiologist, and a heart surgeon are capable of evaluating complete FFR-CT + CT data of patients with multi-vessel disease and/or common trunk disease, to reach an agreement on the type of revascularization to be performed (coronary artery bypass, coronary angioplasty), even better than that achieved by comparing only ICA images [107]. The PACIFIC (Prospective Head-to-Head Comparison of Coronary CT Angiography, Myocardial Perfusion SPECT, PET, and Hybrid Imaging for Diagnosis of Ischemic Heart Disease using Fractional Flow Reserve as Index for Functional Severity of Coronary Stenoses) FFR-CT sub-study compared CCTA, FFR-CT, SPECT, and PET against invasive FFR. FFR-CT was superior to anatomical CCTA, SPECT, and PET in terms of sensitivity, more accurate than CCTA and SPECT, and showed higher specificity than SPECT. FFRCT analysis was equivalent to SPECT but inferior to PET for diagnosing myocardial ischemia, suggesting that FFR-CT may have advantage over other noninvasive tests [108]. FFR-CT could also be used as an innovative tool to evaluate the results of medical therapy (e.g., high-dose statins) [77] and help to plan revascularization procedures through simulation of angioplasty on a specific coronary artery plaque (“virtual stenting”) [109]. Two future randomized trials will provide further data on the clinical advantage of FFR-CT in patients with CCS. The PRECISE (Prospective Randomized Trial of the Optimal Evaluation of Cardiac Symptoms and Revascularization) trial will evaluate if an assessment with a combination of risk stratification using the PROMISE Risk Tool with CCTA and selective FFR-CT could improve outcomes over standard care and safely defer further testing in low-risk patients. The DECISION trial will randomize patients between angiography and FFR- or non-hyperemic pressure ratio-guided revascularization vs. an FFR-CT-guided approach involving clinical decision making based on the HeartFlow Planner. 

The presence of myocardial ischemia has already proved to guide myocardial revascularization with adequate accuracy. Recently, Nagel et al. in the MR-INFORM (Magnetic Resonance Perfusion or Fractional Flow Reserve in Coronary Disease) study demonstrated a noninvasive imaging approach with stress perfusion CMR to be noninferior to FFR in planning appropriate invasive procedures with respect to future cardiac events. The imaging-based approach additionally lead to fewer referrals for ICA, though many patients presented with risk factors and high pre-test probability of CAD [110].

### 6.2. Stress CTP

Under resting conditions, the coronary circulation maintains a constant pressure gradient thanks to an efficient control of the tone of the arterioles and of the microcirculation, able to provide adequate myocardial perfusion even under critical vessel lumen reductions (auto-regulation). However, in the presence of a hyperemic stimulus (exercise or pharmacological stressor) and with an obstructive coronary stenosis, such auto-regulation is lost, resulting in a linear correlation between coronary flow and myocardial perfusion, which may become significantly reduced with a progressive expansion from the subendocardial to the subepicardial layers [111,112].

**Figure 4:**
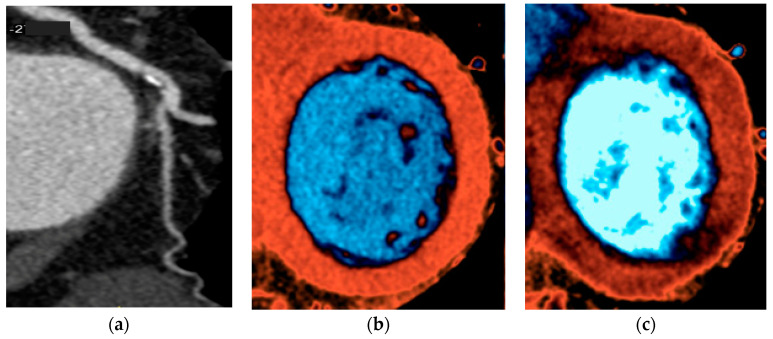
D.R. 45 years-old, female; hypertension; atypical chest pain; CCTA (**a**): fibrocalcific plaque in left circumflex artery with moderate stenosis; stress-CTP perfusion sequences (**c**) matched with rest perfusion sequences (**b**): reversible perfusion defect in basal inferolateral wall. CCTA: coronary computed tomography angiography; CTP: computed tomography perfusion.

Figure 4 shows an example of CTP under rest and hyperemia, unmasking myocardial ischemia.

The performance of CTP requires the evaluation of its passage from the vascular to the myocardial compartment, with attenuation of radiation by the contrast agent proportional to its amount. As a result, reduced density zones, either hypo-enhanced or non-enhanced, represent regions with reduced perfusion in the myocardium. The evaluation of myocardial ischemia is performed after the administration of adenosine during the stress phase, which can eventually either precede or follow the rest phase. In detail, a rest/stress protocol is recommended in cases with lower CAD probability, in order to exclude relevance of disease only when potentially significant lesions are present. In this case, sublingual nitrates and beta-blockers (if heart rate >65 bpm) are first given, followed by a resting anatomical CT scan, along with myocardial perfusion at rest. After a break of about 15 min to allow adequate “wash-out” of contrast medium and premedication, pharmacological stress is given, and finally, myocardial perfusion assessment is performed. On the other hand, if the patient presents a higher risk profile for obstructive CAD, or has been previously revascularized, it may be appropriate to apply the stress/rest protocol, thus avoiding premedication with nitrates and beta-blockers that can mask the presence of ischemia. In the case of a qualitatively optimal stress dataset, myocardial perfusion analysis is possible within a single acquisition, significantly reducing exposure to ionizing radiation [113].

Stress CTP images can be acquired through static and dynamic protocols. In the *static protocol*, a dataset of images through entire cardiac volume during contrast passage is acquired. Once it is acquired, the evaluation is performed by analyzing the multiplanar cardiac projections (multiplanar reconstruction of long axis and short axis), using an increased slice thickness (between 4 and 10 mm), and then comparing the attenuation of myocardial regions suspected for ischemia with the density of an area of remote myocardium. Moreover, adequate analysis of images is undergone with optimal post-processing parameters [114].

Hypoenhanced areas correspond to either myocardial ischemia or scarring; in detail, when the perfusion defect is present in both rest and stress images, it is suggestive of infarction, while a stress only hypoperfusion is mainly due to inducible ischemia [115]. Additionally, true perfusion defects are persistent through various heartbeats and spread through coronary territories, differently from artifacts [116].

The evaluation of static CTP images is purely qualitative. In the past, a semi-quantitative assessment was introduced, characterized by the determination for each heart segment of an index called TPR (transmural perfusion ratio), in Hounsfield units [117]. However, this type of analysis tends to lengthen the reporting time without increasing the diagnostic accuracy in respect to the purely qualitative approach, and is therefore not routinely used [118]. This is a functional evaluation characterized by high diagnostic accuracy, with a limited increase in overall exposure to ionizing radiation [9].

In contrast, the *dynamic protocol* is characterized by the acquisition of multiple datasets, following the kinetics of contrast in the cardiac chambers, deriving time-attenuation curves (TACs). From TACs, different methods can give a value of myocardial blood flow (MBF) for each myocardial segment, usually expressed as mL/100 g/min, which is proportional to myocardial contrast deposit [119,120].

Technically, two types of dynamic protocols are available, proposed by different manufacturers, and related to hardware aspects of the scanner (detector length), the “shuttle-mode”, and the “whole-heart coverage” mode. The most interesting advantage of dynamic stress CTP is its quantitative approach, which makes the report less operator-dependent and more reproducible. Thus, this allows better recognition of multivessel obstructive coronary disease or microcirculation dysfunction. The radiation exposure of dynamic stress CTP varies between 8–9 mSv for “shuttle-mode” and 5 mSv for “whole-heart coverage mode”.

CTP among patients affected by CCS was assessed against functional imaging including SPECT, PET, and CMR [121], as in the CORE320 study [122]. In the PERFECTION (Stress Computed Tomography Perfusion Versus Fractional Flow Reserve CT Derived in Suspected Coronary Artery Disease) study, Pontone et al. revealed a diagnostic accuracy of 93% and 91% in a per-vessel and per-patient analyses of combination of static CTP with CCTA by using a latest generation CT scanner, which was significantly higher than in a CCTA alone strategy [109].

This technique can also improve the performance of CCTA in patients with previously implanted metallic stents, as recently demonstrated by the ADVANTAGE (Additional Diagnostic Value of CT perfusion over coronary CT Angiography in stented patients with suspected in-stent restenosis or coronary artery disease progression) study. This study enrolled 150 patients previously treated with PCI who underwent both stress CTP + CCTA and ICA, suggesting that CTP significantly improves the diagnostic accuracy of CCTA alone [123]. The dynamic CTP (“shuttle-mode” technique) added to the anatomical evaluation, increases the per-vessel specificity and positive predictive value of CCTA [124]. Similar findings were also reported with more potent scanners but with higher radiation exposure (19.4 mSv) [125]. Similar to the CRESCENT II (Comprehensive Cardiac CT Versus Exercise Testing in Suspected Coronary Artery Disease 2) trial (randomized multicenter trial which compared the use of a “functional” strategy, mostly exercise ECG and SPECT, with the use of a “CT” strategy with possible stress CTP in the case of stenosis >50% at cardiac CT), Lubbers et al. highlighted that in suspected stable ischemic heart disease, the CT strategy with possible CTP was more effective (fewer diagnostic investigations; more ICA followed by revascularization for the presence of significant stenosis) and equally safe (same number of clinical events; modest increase in overall radiation exposure) as compared to a functional approach [126]. A meta-analysis included 13 studies and 482 patients with adenosine as the most utilized hyperemic agent and dual-source CT as the most utilized scanner type (69%). Dynamic CTP showed good diagnostic performance compared to different reference standards, including invasive FFR. Sensitivity and specificity approximately around 85% and 93% were oulined [127]. Results obtained in recent studies demonstrated that dynamic CTP may have a prognostic role over anatomical evaluation and FFR-CT [128]. The use of latest generation multidetector-CT scanners (with at least 64 rows) with adequate temporal and spatial resolution (75 ms and 0.23 mm, respectively) is fundamental. This is crucial for proper display of the heart muscle and giving adenosine, the main vasodilator agent used in the stress phase which usually induces an increase in heart rate of about 20 bpm from resting conditions.

Currently, there are few publications that have compared FFR-CT with stress-CTP. Pontone et al. compared myocardial stress perfusion with FFR-CT analysis (provided by HeartFlow), showing how the integrated coronary CT + FFR-CT protocol and the integrated coronary CT + static CTP protocol overlap in terms of diagnostic appropriateness, in the per vessel (CT + FFR-CT 92% and CT + CTP 94%) and per patient analysis (CT + FFR-CT 87% and CT + CTP 92%) [114]. Specificity and positive predictive value were slightly in favor of stress CTP (per vessel analysis, CT + CTP 95% and 87% vs. CT + FFR-CT 94% and 84%, respectively).

Current scientific data show how integration of anatomical and functional analysis using a single method is a safe, feasible, accurate, and reproducible strategy, both with FFR-CT and stress CTP.

## 7. Specific Roles of Cardiac CT: TAVI Planning and Follow-Up of Heart Transplantation

### 7.1. Cardiac CT and TAVI

From the early 2000, treatment of severe aortic stenosis has been revolutionized through initiation of transcatheter aortic valve implantation (TAVI) [129]. A not negligible part in the pre-procedural planning of TAVI relies on cardiac CT, which currently represents the gold-standard imaging test to allow a comprehensive, three-dimensional view of the heart, the aortic valve, the aorta, and its branch vessels. Many CCS patients suffer from multiple CVD risk factors, which also predispone to endothelial degeneration, fibrous thickening, and calcifications of valve tissues, thus favoring advanced valvular disease. Moreover, these are often high-risk patients, in whom an alternative to cardiac surgery, such as TAVI, is preferable.

Cardiac CT protocol for TAVI relies on a retrospective ECG-gated CCTA acquisition of the ascending aorta for evaluation of prothesis size and type, immediately followed by a non-ECG-gated CT acquisition of the peripheral vessels to explore access sites [130]. Of note, a single contrast volume is injected (maximal 100 mL). Lower contrast agent doses are needed in patients with renal function impairment, eventually accepting reduced highlighting of all vessel lumens, though at least vessel walls should be clearly appreciated. Fundamental target in the post-processing analysis is to provide accurate measurements of the aortic root, in particular the anulus diameters (short and long) and its planimetry. The degree of calcifications should also be reported, since severe calcifications may favor TAVI failure after implantation [131]. Assessment of peripheral vessels should report tortuosity and presence of vessel lesions such as calcified or noncalcified plaques [132].

After TAVI, CT may assess early and late complications, such as prothesis leakage, thrombus formation, and prothesis migration.

Performance of cardiac CT in patients scheduled for TAVI is rapid, relatively safe, and allows a comprehensive, detailed assessment of cardiovascular anatomy for optimal procedure planning and assess potential TAVI-related risks.

### 7.2. Cardiac CT and Heart Transplantation

Heart transplant patients require strict follow-up to assess for graft early and late complications, such as graft failure and cardiac allograft vasculopathy (CAV). From histological data, CAV affects mainly the intimal layer of both small and epicardial coronary branches, which becomes thickened, leading to vessel wall negative remodeling and ultimately plaque formation with lumen cross-sectional area reduction [133]. In order to detect CAV, commonly routine ICAs are performed, eventually with intravascular imaging. However, regular screening with invasive methods may be complicated by vascular lesions, as well as ischemic and hemorragic damage [134]. Coronary CT angiography may be performed as an alternative to ICA, and demonstrated adequate specificity (80%) and sensitivity (95%) in detecting CAV, as outlined in recent studies that validated it against intravascular ultrasound (IVUS) [135]. Specific CCTA protocols have been tested, for example, with single-heartbeat high-pitch acquisitions, which resulted feasible in transplant patients with good image quality at an even lower radiation exposure than standard tests [136]. This is an important topic, since CAV detection requires seriated and regularly scheduled screening.

Since CAV is mostly a progressive disease without overt coronary stenosis in the early stages, a combination of CCTA with direct plaque quantification methods may add further diagnostic accuracy in CAV surveillance. A recent study by Miller et al. involving quantification of noncalcified plaque volume demonstrated a higher sensitivity and specificity by this combined approach and suggested performance of advanced plaque analysis in all studies where qualitative view has not evidenced relevant plaques [137].

In a study by Oebel et al., a combined protocol of CCTA plus stress CTP was tested to unmask both anatomical and functional CAV-related consequences, and revealed a very high accuracy on a per-patient basis when compared to ICA and stress-CMR [138].

Regarding these specific roles of cardiac CT, some aspects would be remarkable to implement its use in clinical practice. For example, in the context of TAVI, latest technological advances are crucial to perform high-quality images even at not optimally controlled heartrates, since often administration of beta-blockers in elderly patients is not possible and rhythm disturbances are quite frequent. The definition of patient-tailored protocols, which incorporate renal impairment as well as clinical characteristics such as age, gender, CV comorbidities, and risk factors is also of paramount importance.

For orthotopic heart transplant patients, due to its wide availability, rapid performance, and safety, cardiac CT may represent a valuable alternative to ICA in the follow-up. However, larger prospective studies are needed to confirm its usefulness and definitively introduce it in a regular surveillance program for CAV.

## 8. Conclusions

Diagnosis and management of CCS rely on several noninvasive diagnostic modalities. CCTA has provided a rapidly growing pathway in the landscape of noninvasive diagnostic imaging, from evaluation of coronary calcium burden and anatomical stenosis to advanced plaque imaging with functional assessment of hemodynamically significant lesions and myocardial ischemia. At the same time, recent technological progress has allowed significant advantages with improvement of image quality, as well as temporal and spatial resolution, with significant reduction in overall radiation exposure. Due to its high feasibility and safety, CCTA is an appealing first line diagnostic noninvasive approach in CCS patients.

## Figures and Tables

**Table 1:** Chronologic and technical evolution of multi-slice computed tomography (CT) scanners.

Year	Detector Rows	Detector *z*-axis Resolution (mm)	Detector *z*-axis Coverage (mm)	Temporal Resolution (ms)	Gantry Rotation Time (ms)
1998	4	1–1.25	20	400	500–800
2001	16	0.5–0.75	24	190–250	380–500
2004	64	0.625	40	175	330–400
2007–2008	256–320	0.5–0.625	160	140–175	280–350
2012	640	0.5	160	137	275

**Table 2:** Radiation exposure and presumed risk of cardiac computed tomography (CT) techniques.

CT Modality	Effective Dose (mSv)	Additional Risks
CACS	1.0–1.5	-
CCTA	<1.0–13.5	Contrast-related, Beta-blockers/Nitroglycerine
FFR-CT	<1.0–13.5	Contrast-related, Beta-blockers/Nitroglycerine
Stress-CTP	2.5–21.6	Contrast-related, Beta-blockers/Nitroglycerine, Adenosine

CT: computed tomography; CCTA: coronary computed tomography angiography; FFR-CT: fractional flow reserve derived from computed tomography; CTP: computed tomography perfusion.
